# Catalytic synthesis of (*E*)-α,β-unsaturated esters from aldehydes and 1,1-diethoxyethylene

**DOI:** 10.3762/bjoc.5.3

**Published:** 2009-01-30

**Authors:** Rubén Manzano, Lidia Ozores, Andreas Job, Lars Rodefeld, Benjamin List

**Affiliations:** 1 Max-Planck-Institut für Kohlenforschung, Kaiser-Wilhelm-Platz 1, 45470 Mülheim an der Ruhr, Germany; 2 Saltigo GmbH, 51369 Leverkusen, Germany; 3 Bayer CropScience AG, 40789 Monheim, Germany

**Keywords:** 1,1-diethoxyethylene, α,β-unsaturated esters

## Abstract

A practical and high yielding synthesis of α,β-unsaturated esters from aldehydes and 1,1-diethoxyethylene was developed.

## Introduction

**Scheme 1:**
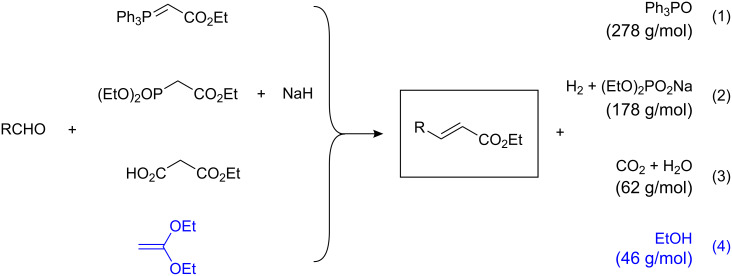
Conversions of aldehydes into α,β-unsaturated esters.

A number of reactions transforming aldehydes into α,β-unsaturated esters have been developed and especially Wittig reaction variants are widely used [1]. A general problem with these approaches, however, is their unsatisfactory atom economy resulting in significant by-product formation (Scheme 1, eq 1–2). Alternatives have been suggested but a solution that is both as general and efficient as the Wittig procedures and satisfactory with respect to the demand for atom economy is still needed [2–22]. We have recently developed a new approach based on the Knoevenagel condensation of readily available and inexpensive malonate half esters with aldehydes which leads to the formation of water and CO_2_ as the only by-products (Scheme 1, eq 3) [23–24]. Here we describe as a new approach, the boronic acid-catalyzed condensation of ketene dialkyl acetal with aldehydes to furnish α,β-unsaturated esters in good yields and reliably high (*E*)-stereoselectivities (Scheme 1, eq 4). Although previously attempted under thermal conditions with poor success, our catalytic reaction is entirely new [25].

We reasoned that the reaction of readily available and inexpensive ketene diethyl acetal [26] or ketene dimethyl acetal (available from Aldrich) with aldehydes upon treatment with a suitable reagent or catalyst could readily lead to the corresponding unsaturated ester and ethanol or methanol as the only by-product. The use of ketene dialkyl acetals for organic transformations is infrequent [27]. To the best of our knowledge, they have been employed in [2+2] cycloadditions [28–30], Diels-Alder processes [31] and reactions with alcohols [32] and *N*-formyl imines [33], to afford orthoesters and *N*-formyl-β-aminoesters respectively.

## Results and Discussion

Initial experiments revealed that Brønsted acids such as acetic acid indeed catalyze the newly designed transformation. After screening various catalysts and different reaction conditions, we found that boronic acids [34], especially 2,4,5-trifluorophenylboronic acid, proved to be quite active catalysts of the condensation of benzaldehyde with ketene diethyl acetal (Table 1). Brønsted acids are also effective but this particular boronic acid, which possesses both Lewis acidity and Brønsted acidity, was the most active catalyst and gave the highest yields of the corresponding α,β-unsaturated ester. The optimized procedure involved the slow addition of 1,1-diethoxyethylene to benzaldehyde in the presence of a catalytic amount (5 mol%) of 2,4,5-trifluorophenylboronic acid at 50 °C in MTBE, affording the corresponding α,β-unsaturated ester in 98% yield as the pure (*E*)-isomer.

**Table 1:** Selected results for catalyst screening.

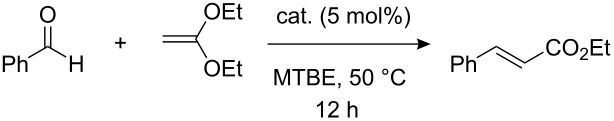		
Entry	Catalyst	Yield^a^

1	2,4,5-C_6_H_2_F_3_-B(OH)_2_	98
2	B(OH)_3_	71
3	*p*-TsOH·H_2_O	86
4	C_6_F_5_-B(OH)_2_	86
5	CF_3_CO_2_H	82

^a^Yield of isolated product

The procedure proved general and both aromatic as well as aliphatic aldehydes can be utilized (Table 2). For aromatic aldehydes the yields are typically very high (93–98%, entries 1–5). Cinnamaldehyde, as an example of an unsaturated aldehyde gave the desired product in 83% yield (entry 6). The unsaturated esters derived from branched aliphatic aldehydes were isolated in 80–89% yield (entries 7–8). Even α-unbranched, linear aldehydes furnished the expected product (entries 9–10). However, in these cases, the yields are slightly lower (61–64%). In addition, 1,1-dimethoxyethylene has also been tested and was found to give the corresponding methyl esters analogously.

**Table 2:** Olefination of different aldehydes.

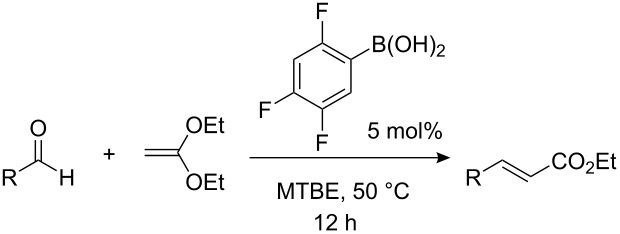		
Entry	R	Yield^a^

1	*p*-MeO-Ph	94
2	Ph	98
3	*p*-Br-Ph	97
4	1-naphthyl	93
5	*p*-MePh	97
6	PhCH=CH	83
7	Cy	89
8	iPr	80
9	*n*-C_4_H_9_	64
10	*n*-C_6_H_13_	61

^a^Yield of isolated product

**Scheme 2:**
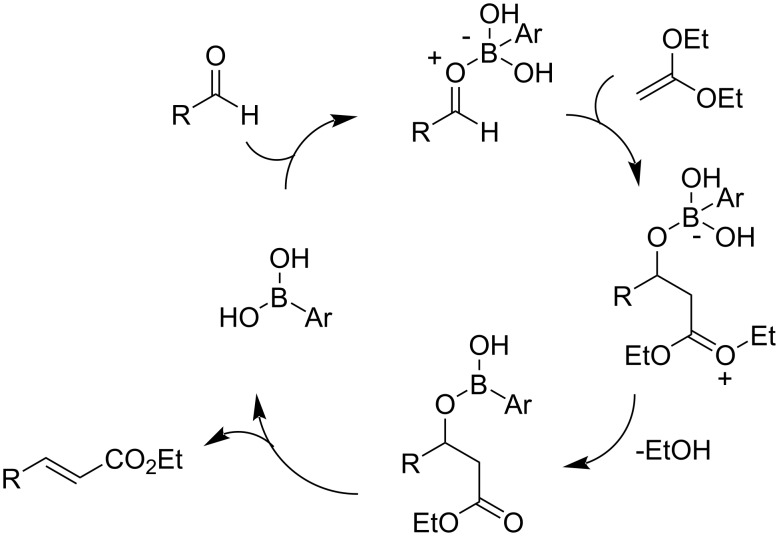
A plausible mechanism.

Despite the absence of any supporting evidence, we envision a plausible mechanism that explains the peculiar effectiveness of boronic acids as catalysts (Scheme 2). Accordingly, the boronic acid functions as a Lewis acid activating the aldehyde but also as a hydroxide donor facilitating the departure of ethanol from an activated intermediate. Clearly, alternative mechanisms may be proposed.

## Experimental

A solution of benzaldehyde (500 mg, 4.7 mmol) and 2,4,5-trifluorophenylboronic acid (0.05 equiv) in MTBE (2.5 mL) was prepared. After heating the reaction mixture to 50 °C, a solution of ketene diethyl acetal (4 equiv) in MTBE (2.5 mL) was added dropwise over 2 h, and the reaction was stirred at the same temperature for 12 h. The solvent was removed under vacuum, and the crude material was purified by flash chromatography to afford the pure product (silica gel, hexane/EtOAc 95/5).
